# Indole-3-carbinol inhibits nasopharyngeal carcinoma cell growth *in vivo* and *in vitro* through inhibition of the PI3K/Akt pathway

**DOI:** 10.3892/etm.2014.1696

**Published:** 2014-04-25

**Authors:** CHENG-GANG MAO, ZE-ZHANG TAO, ZHE CHEN, CHEN CHEN, SHI-MING CHEN, LI-JIA WAN

**Affiliations:** 1 Department of Otolaryngology-Head and Neck Surgery, Renmin Hospital of Wuhan University, Wuhan, Hubei 430060, P.R. China; 2 Department of Otolaryngology-Head and Neck Surgery, The Affiliated Jingzhou Central Hospital of Tongji Medical College, Huazhong University of Science & Technology, Jingzhou, Hubei 434020, P.R. China

**Keywords:** nasopharyngeal carcinoma, indole-3-carbinol, apoptosis, phosphatidylinositol 3-kinase/Akt pathway

## Abstract

Indole-3-carbinol (I3C) is an active component of cruciferous vegetables and has been shown to markedly inhibit the growth of a variety of tumors. However, the role of I3C in nasopharyngeal carcinoma (NPC) remains unclear. Thus, the aim of the present study was to investigate the inhibition of NPC cells by I3C *in vitro* and *in vivo*. The human CNE2 NPC cell line was treated with various concentrations (0, 100, 200 and 300 μM) of I3C and analysis of cell proliferation after 0, 24, 48 and 72 h, apoptosis after 48 h and expression levels of phosphatidylinositol 3-kinase (PI3K)/Akt pathway-associated proteins *in vitro* was performed. BALB/c nude mice were divided into the following groups: Prevention, treatment and control. *In vivo*, all the nude mice were inoculated with CNE2 NPC cells and the mice in the prevention and treatment groups were administered a diet containing 0.5% I3C prior to and following inoculation, respectively. The tumoricidal effect of I3C was investigated in the nude mice. After eight weeks, the expression levels of PI3K/Akt pathway-associated proteins were analyzed in the tumors from the nude mice in each group. The results demonstrated that with increasing I3C concentrations, cell proliferation decreased and apoptosis increased significantly. In addition, the expression levels of PI3K/Akt pathway-associated proteins decreased. In the animal experiments, the prevention and treatment groups developed smaller tumors and the expression levels of PI3K/Akt pathway-associated proteins were reduced when compared with those in the control group. In addition, very few changes to the heart, liver and kidney tissues were observed with hematoxylin and eosin staining in all the groups. Therefore, the results of the present study indicated that I3C inhibited the growth of NPC cells and induced apoptosis effectively *in vivo* and *in vitro*. The underlying mechanism may be that I3C suppresses the PI3K/Akt pathway.

## Introduction

In nasopharyngeal carcinoma (NPC), tumors originate from the epithelial cells that cover the surface of the nasopharynx. The incidence of NPC is particularly high in Chinese and Tunisian populations (1). At diagnosis, 70% of patients have locally advanced, non-metastatic stage III or IV NPC (2,3). Despite novel advances in radiotherapy, chemotherapy and gene-targeting agents, the overall survival rate of patients with aggressive phase NPC remains low (4).

Although a number of chemotherapeutic drugs are available for the treatment of cancer, a highly effective and less toxic approach for treating NPC is required. One potential resource for a new generation of therapeutic agents targeting the prevention and treatment of NPC may be natural substances. For example, epidemiological studies have indicated that the intake of broccoli, cauliflower and other cruciferous vegetables can significantly reduce the incidence of numerous types of cancer, including bladder, pancreas, colon, lung and stomach (5,6). Indole-3-carbinol (I3C) has been identified as an important tumoricidal component in cruciferous vegetables, particularly in members of the genus *Brassica* (7,8). A number of studies have demonstrated that I3C induces cell cycle arrest at the G1 phase in cancer cells (9,10), promotes apoptosis (9–11) and prevents tumor invasion and metastasis (11). I3C has been shown to promote cell cycle arrest by downregulating cyclin-proteins, including cyclin D1 and E. In addition, IC3 has been hypothesized to induce apoptosis by mechanisms that depend on the downregulation of the antiapoptotic genes Bcl-2, Bcl-xL and survivin, as well as by enhancing the expression of Bax and by functionally activating caspase-3 and 9 (10–13).

The phosphatidylinositol 3-kinase/serine-threonine kinase (PI3K/Akt) signaling pathway is involved in the activation of antiapoptotic mechanisms, glucose metabolism and protein synthesis, all of which affect cell growth and proliferation (14,15). Abnormal activation of the PI3K/Akt pathway has been identified in a number of malignancies. In addition, it is becoming apparent that tyrosine kinase-mediated activation of PI3K may be important; for example, in the context of phosphorylated tyrosine kinases interacting with the p85 subunit or mutated Ras binding to PI3K, leading to the activation of PI3K (4).

In addition, somatic mutations may also play an important role. For example, a mutation in the PTEN tumor-suppressor gene may disrupt the ability of PTEN to switch off the PI3K pathway. In addition, PIK3CA gene mutations have been shown to occur in ~30% of epithelial tumors. The abnormal activation of PI3K and somatic mutations may collectively drive the uncontrolled growth and cell proliferation observed during the development of tumors, including ovarian, breast, pancreatic, lung and colon cancer (14,16,17).

In the present study, the application of I3C for the treatment of NPC was investigated *in vivo* and *in vitro*, as well as the potential tumoricidal mechanism. In addition, the preventive and treatment effects of I3C against NPC were explored.

## Materials and methods

### Cell cultures and reagents

The poorly differentiated CNE2 NPC human cell line was purchased from The Cell Bank of Type Culture Collection of Chinese Academy of Sciences (Shanghai, China) and maintained in RPMI-1640 medium containing 10% fetal bovine serum (HyClone Laboratories, Inc., Logan, UT, USA), 100 U/ml penicillin and 100 mg/ml streptomycin at 37°C in a humidified atmosphere with 5% CO_2_. I3C was purchased from Sigma-Aldrich (St. Louis, MO, USA) and specific pathogen-free (SPF) female BALB/c nude mice (age, 4 weeks; weight, 18–20 g) were obtained from Beijing HFK Bioscience Co., Ltd. (Beijing, China).

### Cell treatment

I3C was dissolved in dimethyl sulfoxide (DMSO) to reach a stock concentration of 1 M, which was subsequently diluted to concentrations of 100, 200 and 300 μM for use in the experiments. The final concentration of DMSO in the I3C preparations was <0.5%. In order to demonstrate that the final concentration of DMSO did not affect the NPC cells, CNE2 cells cultured in complete medium alone, without exposure to I3C (as the untreated control) or DMSO (<0.5%), were used as the negative control group (0 μM).

### Cell proliferation and cytotoxicity assays

CNE2 cells in complete culture medium were seeded into 96-well plates at a density of 1×10^3^ cells/ml. The cells were stimulated with I3C at concentrations of 0, 100, 200 or 300 μM in replicates of six wells per I3C concentration. An additional six wells contained culture medium alone as a blank control. After 0, 24, 48 and 72 h, cells were treated with 10 μl cell counting kit (CCK)-8 reagent (Dojindo, Kumamoto, Japan) and incubated at 37°C for 1 h. An automatic microtiter plate reader was set to zero using the blank control wells and the absorbance of the wells was measured at a wavelength of 450 nm. The percentage of cell proliferation inhibition was calculated using the following formula: Cell proliferation inhibition (%) = (1 - average absorbance of the experimental group/average absorbance of the control group) × 100.

### Flow cytometric analysis of apoptotic cells using annexin V-fluorescein isothiocyanate (FITC) and propidium iodide (PI)

CNE2 cells in complete culture medium containing 0, 100, 200 or 300 μM I3C, were seeded into 6-well plates. Additional wells were seeded with culture medium alone as an untreated control group and each group was seeded in triplicate. After 48 h, the cells were harvested by centrifugation, resuspended in binding buffer and successively incubated with 5 μl annexin V-FITC and 5 μl PI (Multi Sciences Biotech Co., Ltd., Hangzhou, China) at room temperature for 15 min. Apoptosis was determined by flow cytometric analysis using a FACSCanto II flow cytometer (BD Biosciences, San Jose, CA, USA). To construct the flow cytometric dot plot, annexin V staining was set as the horizontal axis and PI staining was set as the vertical axis. Mechanically damaged cells were located in the upper left quadrant, apoptotic or necrotic cells were located in the upper right quadrant, dual negative and normal cells were present in the lower left quadrant and early apoptotic cells were observed in the lower right quadrant of the flow cytometric dot plot.

### Western blot analysis

After 48 h of treatment with various concentrations of I3C, cells were lysed in radioimmunoprecipitation assay (RIPA) buffer to extract total cellular protein. Protein concentrations were determined using the bicinchoninic acid (BCA) quantitative method and 30-μg samples of each protein were resolved by SDS-PAGE. Next, the protein bands were transferred to a nitrocellulose membrane. Following protein transfer, the membrane was blocked for 1 h in the presence of 5% skimmed milk proteins and incubated at 4°C overnight with primary antibodies (Cell Signaling Technology, Inc., Beverly, MA, USA) targeted against PI3K p110α, PI3K p85, phosphorylated-Akt (p-Akt), Akt, phosphorylated-glycogen synthase kinase (p-GSK)3-β, GSK3-β and GAPDH (as an internal reference housekeeping protein). The blots were then incubated with a secondary antibody at room temperature for 1 h and specific protein bands were visualized using an enhanced chemiluminescence assay kit (Pierce Biotechnology, Inc., Rockford, IL, USA).

### Animal selection and breeding

SPF female BALB/c nude mice (age, 4 weeks; weight, 18–20 g) were used. All animal husbandry procedures and experiments were conducted under SPF conditions. Prior to the experiment, animals were provided with a conventional diet for 1 week. Following this adaptive feeding, I3C was incorporated into the conventional feed at a ratio of 0.5% (w/w). All experimental procedures in the study were approved by the local Ethics and Animal Care and Use Committee of Wuhan University (Wuhan, China).

### Establishing tumor-bearing animal models in BALB/c nude mice

A total of 24 female BALB/c mice were randomly assigned to pretreatment, treatment and control groups (n=8 mice per group). CNE2 cells were cultured to a confluence of 80–90%, harvested by treatment with trypsin and, following centrifugation, were resuspended in plasma. The cells, at a density of 1×10^7^ cells/ml, were then adoptively transferred into the mice by the administration of 200-μl subcutaneous injections of the cell suspension in plasma into the right side of the back of the nude mice. The pretreatment group received a conventional diet supplemented with I3C at a ratio of 0.5% (w/w) for 2 weeks prior to the adoptive CNE2 cell transfer; following injection of the tumor cells, the mice in this group received conventional feed without supplementation. The treatment group received a conventional diet supplemented with I3C at a 0.5% (w/w) ratio following the transfer of CNE2 cells. The mice continued to receive the I3C supplemented diet until the experiment had reached the endpoint at week 8. Mice in the control group were fed conventional feed without supplementation. For statistical analysis, at the end of each week following the adoptive transfer of CNE2 cells, the maximum (a) and minimum (b) diameters of the tumor were measured and the volume was calculated using the formula: Volume = 1/2ab^2^. After 8 weeks, the mice were sacrificed by cervical dislocation and specimens were collected from the tumor, heart, liver and kidney.

### Protein extraction and expression assays

Tumor specimens from the sacrificed mice were cut into regularly sized sections and minced using a tissue homogenizer. Immediately following this preparation, the cells were lysed in RIPA buffer and the extracted proteins were quantified using the BCA method. Western blot analysis was applied to detect the specific protein expression levels of PI3K, Akt and GSK3-β.

### Morphological observation of the heart, liver and kidney tissue sections

Biopsy specimens of the heart, liver and kidney obtained from the nude mice were fixed in 10% formalin, dehydrated and embedded in wax. The samples were sectioned into 5–8-μm slices and pasted onto slides. Next, the specimens were dewaxed and stained with hematoxylin and eosin (H&E) for final histological evaluation using a standard light microscope.

### Statistical analysis

Statistical analysis was conducted using SPSS statistical software, version 16.0 (SPSS, Inc., Chicago, IL, USA) for Microsoft Windows. Results are expressed as the mean ± SD. Differences between multiple groups were compared by analysis of variance and P<0.05 was considered to indicate a statistically significant difference.

## Results

### I3C treatment markedly inhibits NPC cell growth

**Figure 1:**
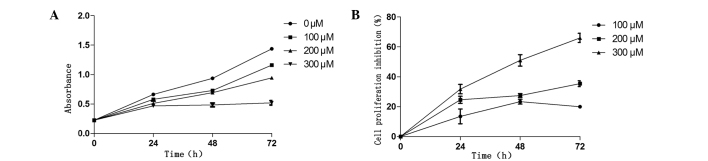
I3C affects CNE2 cell proliferation. (A) CNE2 cells were treated with I3C (0, 100, 200 or 300 μM) for 24, 48 and 72 h. NPC cell proliferation was detected using the CCK-8 method and absorbance values are expressed as mean ± SD. All P<0.05, vs. negative control (0 μM). (B) Association between the percentage of NPC cell proliferation inhibition and the concentration of I3C. I3C, indole-3-carbinol; NPC, nasopharyngeal carcinoma; CCK-8, cell counting kit-8.

Following treatment of the CNE2 cells with I3C at concentrations of 100, 200 and 300 μM for various times (24, 48 and 72 h), concentration- and time-dependent inhibition of cell growth was observed. Statistically significant differences in cell growth were observed between the treated and negative control groups (P<0.05; Fig. 1A). In the group treated with 100 μM I3C, at the indicated time points of 24, 48 and 72 h, the percentage of CNE2 cell proliferation inhibition was 16.1, 20.2 and 18.8%, respectively. In addition, in the group treated with 200 μM I3C, the percentage of cell proliferation inhibition at 24, 48 and 72 h was 22.8, 26.0 and 34.2%, respectively. In the 300 μM I3C group, the percentage of cell proliferation inhibition at 24, 48 and 72 h was 29.4, 48.4 and 63.9%, respectively (Fig. 1B). Therefore, inhibiting the proliferation of CNE2 NPC cells with I3C was highly concentration-dependent.

### I3C treatment induces NPC CNE2 cell apoptosis

**Figure 2:**
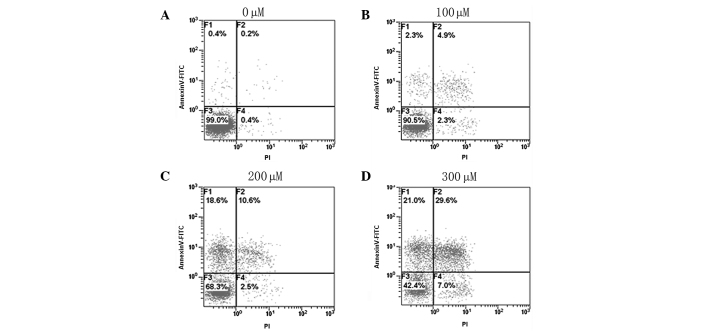
Fluorescence-activated cell sorting analysis of the effect of I3C on NPC cell apoptosis. CNE2 cells were treated with (A) 0, (B) 100, (C) 200 or (D) 300 μM I3C for 48 h and analyzed with annexin V-PI double staining to detect the apoptotic rates. P<0.05, for the 200 and 300 μM groups vs. the negative control (0 μM). NPC, nasopharyngeal carcinoma; I3C, indole-3-carbinol; PI, propidium iodide.

Analysis of CNE2 cell apoptosis with annexin V-PI double staining revealed that after 48 h of treatment with 0, 100, 200 and 300 μM I3C, the apoptotic rates were 0.4±0.25, 2.3±1.35, 18.6±2.32 and 21.0±5.28%, respectively. In addition, when comparing the 100 and 0 μM concentrations, there was no statistically significant difference observed in the rate of apoptosis. However, statistically significant increases were observed in the apoptotic rates of the 200 and 300 μM groups when compared with those in the 0 μM group (P<0.05). Therefore, a concentration of 100 μM I3C induced apoptosis of CNE2 cells, while a concentration of 300 μM increased the proportion of apoptotic cells and exhibited an apoptotic rate >20% (Fig. 2). Thus, the results indicated that the proportion of cells undergoing apoptosis following treatment with I3C was concentration-dependent (Fig. 2).

### I3C treatment significantly reduces the expression levels of PI3K/Akt-associated proteins in CNE2 cells

**Figure 3:**
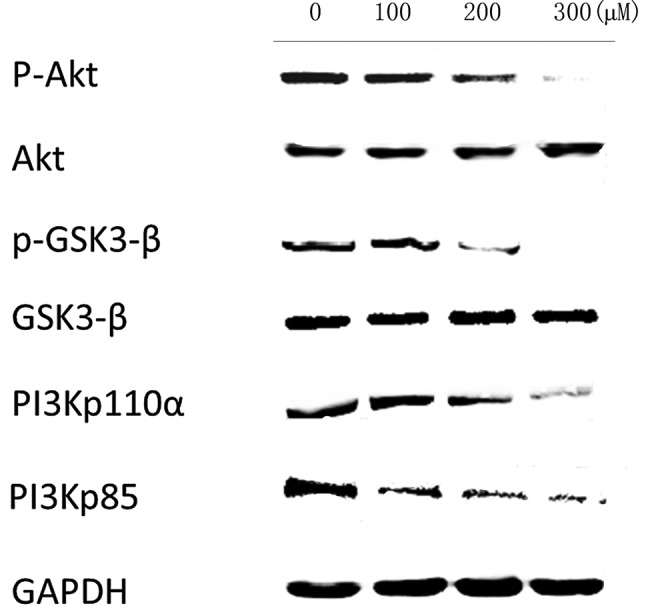
CNE2 cells were treated with 0, 100, 200 and 300 μM I3C, after which the expression levels of key proteins in the PI3K/Akt pathway and downstream proteins were analyzed by western blot analysis. GAPDH served as an internal reference control. PI3K, phosphatidylinositol 3-kinase; Akt, protein kinase B; I3C, indole-3-carbinol.

The expression levels of key signaling proteins involved in the PI3K/Akt pathway and downstream signaling proteins associated with cancer cell proliferation and apoptosis were analyzed. In the untreated control and negative control groups, the protein expression of PI3K p110α, PI3K class III, p-Akt, phosphorylated-c-Raf and GSK3-β was clearly observed. Treatment with I3C markedly decreased the protein expression levels of PI3K p110α, PI3K p85, p-Akt, Akt, p-GSK3-β and GSK3-β when compared with the levels in the untreated groups. In addition, the decreased expression levels of the signaling proteins was identified to be I3C concentration-dependent (Fig. 3).

### Tumoricidal effects of I3C in a BALB/c nude mouse model

The tumoricidal effects of I3C were further investigated in animal model experiments. The control mouse group received a conventional diet only and these mice subsequently developed large tumors. At week 8, the average volume of the tumors was 2,236.7±255.2 mm^3^. By contrast, the mean volumes of the tumors in the I3C treatment and pretreatment groups were 1,523.6±143.6 and 1,135.3±236.2 mm^3^, respectively (P<0.05). These results indicated that I3C significantly inhibited tumor growth (P<0.05). In addition, the administration of I3C prior to tumor transplantation was more effective than I3C administration following tumor transplantation (P<0.05).

### I3C treatment significantly reduces the expression of PI3K/Akt and downstream signaling proteins in the transplanted tumors

**Figure 4:**
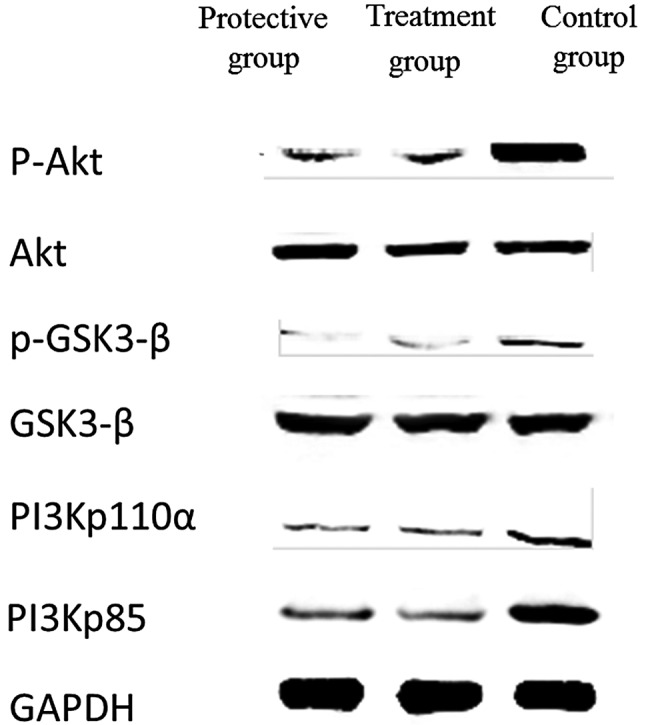
I3C inhibition of tumor growth via the PI3K/Akt pathway in a CNE2 xenograft tumor model. Eight weeks following tumor implantation, the expression levels of PI3K/Akt pathway-associated proteins were analyzed by western blot analysis in the tumor tissues of the protective, treatment and control groups. GAPDH served as an internal reference control. PI3K, phosphatidylinositol 3-kinase; Akt, protein kinase B; I3C, indole-3-carbinol.

Expression levels of proteins associated with the PI3K/Akt pathway and downstream signaling pathways were analyzed in the transplanted tumors. Pretreatment and treatment with I3C was found to be associated with reductions in the expression levels of PI3K p110α, PI3K p85, p-Akt and p-GSK3-β signaling proteins. The lowest expression levels of signaling proteins were detected in the pretreatment group, which was followed by the treatment group. The highest expression levels of signaling proteins were identified in the control group (Fig. 4).

### I3C does not produce harmful side-effects in the heart, liver and kidney of nude mice

**Figure 5:**
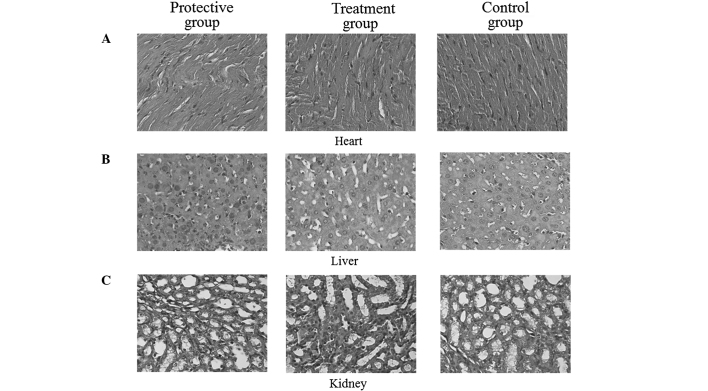
H&E staining of specimens from the (A) heart, (B) liver and (C) kidney of I3C-treated nude mice. Sections from the protective, treatment and control groups were stained with H&E to observe and assess cell morphology and tissue structure (magnification, ×400). H&E, hematoxylin and eosin; I3C, indole-3-carbinol.

Specimens from the heart, liver and kidney obtained from the nude mice were sectioned and stained with H&E. Pathological evaluation revealed that treatment with I3C exerted no adverse effects on the heart, liver or kidney in the nude mice. No tissue damage, including structural degeneration or tissue necrosis, was observed in any of the tissue specimens. These results indicated that supplementing the feed with 0.5% (w/w) I3C did not provoke any toxic side-effects in the examined organs of the experimental mice (Fig. 5).

## Discussion

I3C is extracted from cruciferous vegetables by the hydrolysis of glucosinolates (7). A number of previous *in vitro* studies have demonstrated that I3C causes significant growth inhibition in a variety of tumor cell lines by inducing cell cycle arrest and apoptosis (9,11,13,18,19). However, the role of I3C in NPC has remained largely unexplored. The observations of the present study revealed that I3C significantly inhibited the proliferation of CNE2 cells with an optimal concentration of 300 μM; at this concentration, the inhibition rate of tumor cell proliferation was 29.4, 48.4 and 63.9% observed at 24, 48 and 72 h, respectively. The inhibitory mechanism of I3C involves altering the proliferation of cancer cells and the induction of apoptosis. In the present study, 200 μM I3C was shown to induce an apoptotic effect. In addition, the apoptotic rate increased in an I3C concentration-dependent manner. Rahman *et al* (11) reported that treatment of breast cancer cells with 30–100 μM I3C for 24–72 h induced apoptosis.

The PI3K/Akt pathway is associated with receptor tyrosine kinase signaling pathways. Mutational activation of pathway components may inappropriately activate PI3K and the downstream target proteins, Akt and p-Akt, subsequently resulting in the phosphorylation and activation of mTOR/GSK3, mouse mdm2, Bad and members of the caspase family that collectively play an important role in promoting tumor cell growth, proliferation, suppression of apoptosis, promotion of cellular invasion, tumor metastasis and angiogenesis (20). Abnormalities in the PI3K/Akt signaling pathway and in particular, abnormal phosphorylation of Akt, have been found to be closely associated with the development of tongue carcinoma and head and neck squamous cell carcinomas (17,21).

To further clarify the potential association between I3C-induced apoptosis and the PI3K/Akt signaling pathway in nasopharyngeal cancer, the differential expression of PI3K/Akt pathway-specific signaling proteins and their cognate downstream proteins were analyzed prior to and following I3C treatment. Markedly decreased expression levels of PI3K p110α, PI3K p85, p-Akt and p-GSK3-β were observed in CNE2 cells following treatment with I3C, as compared with the levels in the untreated cells. This observation indicates that the inhibition of NPC cell proliferation and the induction of apoptosis are closely associated with the PI3K/Akt signaling pathway. The results of the present study are in accordance with previous studies of breast and prostate cancer (9,21).

Previous animal studies (17,18) have demonstrated that I3C inhibits the occurrence and development of solid tumors. In addition, previous studies on cervical cancer have revealed that the antitumor mechanism of I3C is partly dependent on an increase in permeability of the tumor cell membrane, activation of caspase-3 and, thus, induction of cancer cell apoptosis (22). When mice with prostate cancer (TRAMP model) were fed diets containing 1% (w/w) I3C over a period of 10 weeks, tumor cell apoptosis was induced (23) and Nrf2, which is involved in a pathway known to regulate antioxidant signaling pathways, was activated. Newfield *et al* (24) reported that the immune response of mice fed I3C may be triggered to produce the antiproliferative agent, 2-hydroxy estrone. The animal experiments of the present study confirmed that I3C effectively inhibited the growth of NPC and that I3C intervention significantly decreased NPC growth in nude mice when compared with the controls. The graft tumor growth rate was slower in the pretreatment group compared with that in the treated mouse group, indicating that I3C effectively prevents the occurrence and development of solid tumors. This observation also indicates that I3C may be considered as an alternative therapeutic approach for the prevention and treatment of NPC.

Consistent with the results obtained from the *in vitro* experiments, in the pretreated and treated nude mice, the expression levels of PI3K p110α, PI3K p85, p-Akt and p-GSK3-β were shown to be significantly downregulated when compared with those in the control group. This observation indicates that abnormal activation of the PI3K/Akt pathway may play a key role in the process of NPC. The animal experiments also confirmed that I3C downregulated the protein expression of PI3K/Akt signaling pathway proteins in the transplanted tumors.

Using I3C to treat MCF10CA1a breast cancer cells and homologous CF10A mammary epithelial cells, Rahman *et al* (11) found that I3C induced apoptosis only in the MCF10CA1a breast cancer cells, indicating that I3C was without risk to nontumor cells. Consistent with this, the current study also confirmed that I3C caused growth inhibition in the transplanted tumors of nude mice only and did not provoke any degenerative harm to the heart, liver and kidney, with low cytotoxicity to normal cells.

In summary, the *in vivo* and *in vitro* experiments of the present study have demonstrated that I3C significantly inhibits NPC proliferation and induces apoptosis. We hypothesize that the observed effects of I3C are likely to be associated with regulation of the PI3K/Akt signaling pathway and downstream protein expression. Due to the effective, non-toxic and natural antitumor properties of I3C, the compound should be considered as a likely preventive and curative candidate for NPC. Additional studies are required to determine the underlying mechanisms by which I3C suppresses NPC at a molecular level, providing a theoretical basis for the tumoricidal and clinical utility of I3C.
